# Exploring the Relationship between Behavioral and Neurological Impairments Due to Mild Cognitive Impairment: Correlation Study between Virtual Kiosk Test and EEG-SSVEP

**DOI:** 10.3390/s24113543

**Published:** 2024-05-30

**Authors:** Dohyun Kim, Yuwon Kim, Jinseok Park, Hojin Choi, Hokyoung Ryu, Martin Loeser, Kyoungwon Seo

**Affiliations:** 1 Department of Applied Artificial Intelligence, Seoul National University of Science and Technology, Seoul 01811, Republic of Korea; dh_kim@seoultech.ac.kr (D.K.); ferrry@seoultech.ac.kr (Y.K.); 2 Department of Neurology, College of Medicine, Hanyang University, Seoul 04763, Republic of Korea; jinspark@hanyang.ac.kr (J.P.); chj@hanyang.ac.kr (H.C.); 3 Graduate School of Technology and Innovation Management, Hanyang University, Seoul 04763, Republic of Korea; hryu@hanyang.ac.kr; 4 Department of Computer Science, Electrical Engineering and Mechatronics, ZHAW Zurich University of Applied Sciences, 8401 Winterthur, Switzerland; loma@zhaw.ch

**Keywords:** virtual reality, brain–computer interface, human cognition, instrumental activities of daily living, steady-state visual evoked potentials

## Abstract

Amnestic mild cognitive impairment (aMCI) is a transitional stage between normal aging and Alzheimer’s disease, making early screening imperative for potential intervention and prevention of progression to Alzheimer’s disease (AD). Therefore, there is a demand for research to identify effective and easy-to-use tools for aMCI screening. While behavioral tests in virtual reality environments have successfully captured behavioral features related to instrumental activities of daily living for aMCI screening, further investigations are necessary to establish connections between cognitive decline and neurological changes. Utilizing electroencephalography with steady-state visual evoked potentials, this study delved into the correlation between behavioral features recorded during virtual reality tests and neurological features obtained by measuring neural activity in the dorsal stream. As a result, this multimodal approach achieved an impressive screening accuracy of 98.38%.

## 1. Introduction

Amnestic mild cognitive impairment (aMCI), positioned between normal aging and Alzheimer’s disease (AD), is characterized by a gradual decline in multiple cognitive domains, such as memory, attention, and executive function [1,2,3,4,5]. Individuals suffering from aMCI face an increased—about 80% [6]—risk of progressing to AD, an irreversible neurodegenerative disorder [3,4,5]. With the growth of pharmacology including Lecanemab [7] and Levetiracetam [8], identifying aMCI at an early stage not only holds promise for decelerating the progression to AD but also presents the potential for a return to normal aging. Hence, precise screening of the early stages of aMCI becomes increasingly important.

In response to the urgent need for early detection of aMCI, a large variety of diagnostic tools such as virtual reality (VR), magnetic resonance imaging (MRI), and electroencephalography (EEG) have been used in recent years. For example, VR shows promise in providing features for aMCI screening through the assessment of instrumental activities of daily living (IADLs), which evaluates cognitive function during complex activities in everyday life [9,10,11,12,13,14,15]. Employing head-mounted displays (HMDs) and hand controllers allows for the easy tracking and recording of behavioral features such as eye and hand movements. Yet, there remain concerns about the feasibility in clinical settings [9,12]. Although MRI allows for the quantitative assessment of brain structure, it could be considered invasive and less suitable for early aMCI screening due to the loud noise [16], potential thermal damage from high-frequency energy [17,18], and the risk of side effects from MRI contrast agents [19]. EEG, on the other hand, can monitor real-time changes in brain activity with temporal accuracy to observe responses to stimuli, albeit with sensitivity to noise [20,21,22,23,24,25]. While various diagnostic tools are employed in clinical settings, each tool presents distinct advantages and limitations.

In recent years, many studies have investigated the integration of diverse diagnostic tools into multimodal models to overcome the above limitations [15,20,26,27]. In this context, combining VR data with EEG–steady state visually evoked potential (SSVEP) emerges as a promising strategy to augment the applicability of VR within clinical settings. Behavioral impairments in aMCI are considered to stem from a compromised dorsal stream of the visual pathway, which governs behavioral responses to visual stimuli [28,29,30,31,32]. EEG-SSVEP is a powerful tool to quantify the impairment of the dorsal stream, as it allows us to capture harmonic responses in the brain’s reaction to periodic visual stimuli applied to a patient’s retina. This is why combining VR and EEG-SSVEP data offers both behavioral and neurological insight, facilitating a holistic assessment of cognitive impairments caused by aMCI. To the best of our knowledge, the research conducted by Xue et al. [26] exhibited a state-of-the-art result in incorporating both VR and EEG for MCI detection. During their research, they combined data from VR HMD with EEG time series data that were recorded during the same experiment. Their investigation, predicting MCI based on features extracted from both VR and EEG, yielded an impressive accuracy of 91.3%. Yet, despite achieving high classification performance, this approach has considerable limitations. First, the absence of consideration for the compromised visual pathway perspective resulted in a lack of insight into the correlation between VR and EEG. The absence of EEG-SSVEP, which allows variations in EEG responses to visual stimuli to be captured, represents a significant gap. Moreover, due to the high sensitivity of EEG sensors to noise [33], the simultaneous use of VR HMD and EEG may entail the loss of meaningful EEG information. This, in turn, might reduce the classification accuracy. Given these considerations, our study was designed to analyze both VR and EEG-SSVEP comprehensively while measuring VR features and EEG-SSVEP features independently.

The main contributions of this paper are threefold. First, we compared and analyzed the behavioral features recorded during a VR test (“virtual kiosk”) and the neurological features from EEG measurements. Here, we compared a healthy control group with aMCI patients. Second, we analyzed the cognitive relationship between behavioral and neurological features. Finally, we demonstrated the potential of multimodal learning by combining both behavioral and neurological features into a single model to assess aMCI.

## 2. Materials and Methods

### 2.1. Participants

We recruited a total of 52 participants, consisting of 24 healthy controls and 28 patients with aMCI, at Hanyang University Hospital from January 2022 to April 2024. The diagnosis of aMCI was confirmed by two experienced neurologists, with 18 and 22 years of expertise, following the diagnostic criteria established by Albert et al. [34]. Inclusion criteria for this study were individuals aged 50 and above who possessed the capability to perceive both visual and auditory stimuli. To exclude cases of aMCI resulting from other conditions, patients with concurrent depressive disorders, vascular dementia, or a history of brain surgery were not considered in our study. Based on these criteria, 4 out of the 28 patients with aMCI were excluded from the final analysis. Consequently, the final analysis included 24 healthy controls and 24 patients with aMCI. All participants provided written informed consent to partake in this research and underwent the Korean Mini-Mental State Examination (K-MMSE) to assess cognitive ability. As shown in Table 1, there were no significant differences in demographics (i.e., gender, age, and years of education) between healthy controls and aMCI patients except K-MMSE (*p* < 0.05). This study received approval from the Institutional Review Board in accordance with the Declaration of Helsinki (HYUH-2021-08-020-004).

### 2.2. Behavioral Features

Behavioral data were collected from a virtual kiosk test—a VR test we developed during our prior study for the assessment of IADL [9]. The experiment was conducted on a high-performance laptop equipped with an Intel i7-12700H processor processor (Intel, Santa Clara, CA, USA), 16GB RAM (Samsung, Gyeonggi-do, Suwon, Republic of Korea), and an NVIDIA GeForce RTX 3080 graphics card (NVIDIA, Santa Clara, CA, USA). To ensure a fully immersive VR experience, participants wore an HTC VIVE Pro Eye (HTC Vive, Taoyuan City, Taiwan) VR headset and held a hand controller on their dominant hand. For safety reasons, all participants were sitting on a chair during the experiments. Embedded eye trackers in the VR headset and two base stations allowed us to track participants’ eye and hand movements simultaneously.

**Figure 1:**
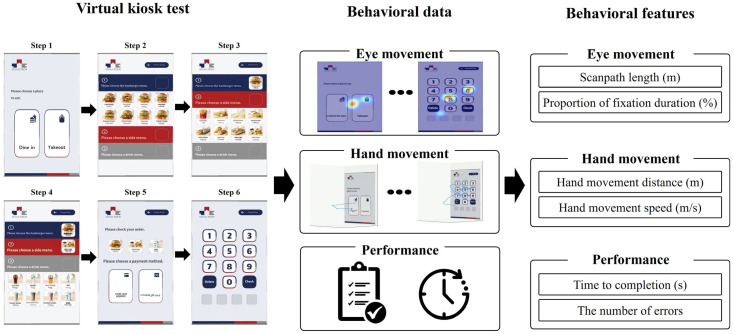
Six behavioral features collected from the virtual kiosk test.

As shown in Figure 1, the virtual kiosk test comprised the following six steps: (1) selecting a place to eat, (2) choosing a burger item, (3) selecting a side item, (4) choosing a drink item, (5) selecting a payment method, and (6) entering a four-digit password. Participants were asked to memorize the following instructions: “The place to eat is a restaurant. Please use the kiosk to order a shrimp burger, cheese sticks, and a Coca-Cola. Use a credit card as the payment method, and the password for payment is 6289”. Prior to the test, participants underwent two practice sessions to become familiar with the VR environment. Participants could halt the test due to dizziness and cybersickness, but all participants completed the test without any interruptions.

During the test, we recorded hand movements, eye movements, and performance data. From these measured data, a total of six behavioral features were derived and utilized in this study. From the eye movement data, two features were extracted: scanpath length, representing the total distance covered by a participant’s gaze during the virtual kiosk test; and proportion of fixation duration, representing the percentage of time a participant focused on the target menu item out of all menu items. From the hand movement data, two additional features were derived: hand movement distance, referring to the total distance of hand trajectory during the virtual kiosk test; and hand movement speed, representing the average speed of hand movements during the test. Finally, from the performance data, we included time to completion, which is the total duration a participant took to complete all six steps of the virtual kiosk test, and number of errors, which represents the total errors made by a participant in the test, as the final two features. This approach allowed us to capture diverse aspects of participant behavior associated with various cognitive functions, such as perception and information processing.

### 2.3. Neurological Features

EEG-SSVEP signals were recorded using the Comet-Plus XL Lab EEG system (Natus Neurology, Middleton, WI, USA) with a sampling rate of 200 Hz. Electrode placement followed the international 10-20 system, incorporating Fp1, Fp2, F3, F4, F7, F8, C3, C4, T3, T4, T5, T6, P3, P4, O1, O2, Fz, Cz, and Pz. Measured data from each electrode were normalized to yield zero average during data processing. Artifacts were eliminated using an automatic and tunable removal algorithm [35], which was implemented in the spkit module in Python 3.9. In this study, we employed the package’s “elimination mode”, utilizing the db4 wavelet, and a β value of 0.1.

**Figure 2:**
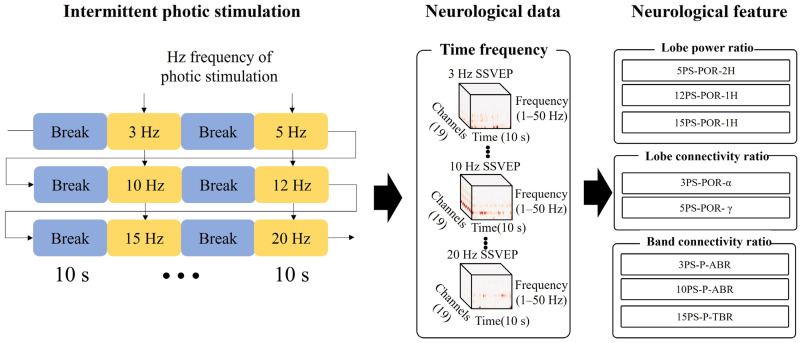
Eight neurological features collected by EEG-SSVEP recording with intermittent photic stimulation.

To investigate the impact of visual stimulation on brain activity, intermittent photic stimulation (IPS) was employed to capture steady-state visual evoked potentials (EEG-SSVEP). Stimulation frequencies of 3, 5, 10, 12, 15, and 20 Hz were utilized, allowing the examination of neural responses and EEG activity specific to each frequency. The IPS session, lasting 120 s, consisted of alternating 10 s periods of photic stimulation at different frequencies and 10 s rest intervals (see Figure 2). Participants were instructed to lie down comfortably in a dimly lit room and close their eyes to induce a state of relaxation and minimize visual distraction while ensuring optimal diffusion of photic stimuli onto the retina [36].

In this study, we investigated the dorsal stream, a crucial element in visual processing for action, utilizing EEG-SSVEP. The dorsal stream‘s response to visual stimuli is characterized by the ratio of signal power in the parietal and occipital lobes(POR). As a first step, this requires the harmonics to be averaged within the stimulation time range for each channel based on time–frequency data. The time–frequency data were obtained using the multi-taper method implemented in MNE-Python, with a sliding time window of 500 ms. The frequency range for the analysis was set from 1 to 50 Hz in steps of 1 Hz. Subsequently, an 8 Hz frequency smoothing algorithm was applied using three tapers. This process was performed using a Fast Fourier Transform with a Hanning taper. Second, channel averaging within each parietal lobe was conducted, with power expressed in dB. Finally, responsiveness to visual stimuli in the dorsal stream was evaluated by computing the parietal-to-occipital ratio.

The equation for power analysis is as follows. In this formula, P represents the power of the harmonic response. x denotes the time–frequency data, e is the electrode index, f refers to the photic stimulation frequency, and n indicates the harmonic number.

(1) $$P_{e,f,n}=\frac{1}{T}\sum_{t=0}^{T}x_{e,f*n}$$

The equation for lobe power (LP) is as follows. In this formula, LP represents the power of the harmonic response for each lobe, calculated by averaging the power values of the electrodes within that lobe. L denotes the set of electrodes belonging to each lobe.

(2) $${LP}_{L,f,n}=\frac{\sum_{e\in L}P_{e,f,n}}{L_{length}}$$

The equation for the lobe power ratio (LPR) is as follows. In this formula, LPR refers to the ratio of lobe power, which compares the harmonic response of each lobe, specifically evaluating the dorsal stream pathway from the occipital to the parietal lobes. P denotes the set of electrodes within the parietal lobe, while O represents the set within the occipital lobe. The photic stimulation frequencies f are 3, 5, 10, 12, 15, and 20 Hz, and n is the number of harmonics where f∗n<50. The electrodes for the parietal lobe are located at P3, P4, C3, C4, Pz, and Cz, while the electrodes for the occipital lobe are located at O1 and O2.

(3) $${LPR}_{f,n}=\frac{{LP}_{P,f,n}}{{LP}_{O,f,n}}$$

The transmission of visual stimuli in the dorsal stream is quantified by the ratio of connectivity in the parietal and occipital lobes (POR), the ratio of θ and β connectivity in the parietal lobe, and the ratio of α and β connectivity in the parietal lobe. As a first step, we used the weighted phase lag index (wPLI) [37] and the weight clustering coefficient developed by Onnela [38] to measure the transmission of visual stimuli in each frequency band. As a second step, we computed the lobe connectivity by averaging connectivity values across channels within each lobe. As a third step, we computed the lobe connectivity ratio to assess the connectivity of the dorsal stream. Finally, we computed the band connectivity ratio based on the θ/β ratio (TBR) and the α/β ratio (ABR) for the parietal lobe.

The equation for connectivity in each EEG channel is as follows. In this formula, C represents the connectivity between each electrode and its neighboring electrodes. f denotes the photic stimulation frequency, b stands for the frequency band, w is the weight matrix of the b band during f Hz photic stimulation obtained through wPLI, e is the electrode index, i and j are variables used to explore the neighboring electrodes of e, and deg signifies the degree.

(4) $$C_{e,f,b}=\frac{\sum_{i,j}{\left(w_{f,b,e,i}w_{f,b,e,j}w_{f,b,i,j}\right)}^{\frac{1}{3}}}{\deg\left(e\right)\deg\left(e\right)-1}$$

The equation for lobe connectivity (LC) is as follows. In this formula, LC represents the connectivity within a lobe, obtained by averaging the connectivity values of the electrodes within that lobe. L denotes the set of electrodes within each lobe.

(5) $${LC}_{L,f,b}=\frac{\sum_{e\in L}C_{e,f,b}}{L_{length}}$$

The equation for the lobe connectivity ratio (LCR) is as follows. In this formula, LCR compares the connectivity between different lobes, specifically evaluating the dorsal stream pathway from the occipital to the parietal lobes. P denotes the set of electrodes within the parietal lobe, while O represents the set of electrodes within the occipital lobe.

(6) $${LCR}_{f,b}=\frac{{LC}_{P,f,b}}{{LC}_{O,f,b}}$$

The equation for the band connectivity ratio (BCR) is as follows. In this formula, BCR is the ratio of connectivity across frequency bands, used to analyze subtle processing differences in visual stimuli in the parietal lobe. It compares the θ and α bands, represented by $b_{1}$, with the β band, represented by $b_{2}$. The photic stimulation frequencies f are 3, 5, 10, 12, 15, and 20. The frequency bands b are defined as θ: 4–8 Hz, α: 8–12 Hz, β: 12–30 Hz, and γ: 30–50 Hz. The electrodes for the parietal lobe are located at P3, P4, C3, C4, Pz, and Cz, while the electrodes for the occipital lobe are located at O1 and O2.

(7) $${BCR}_{f,b_{1},b_{2}}=\frac{{LC}_{P,f,b_{1}}}{{LC}_{P,f,b_{2}}}$$

To enhance understanding, we established three components for naming neurological features: (1) the PS frequency (3, 5, 10, 12, 15, and 20 Hz), (2) the target lobe (parietal lobe, POR), and (3) the specific frequency ranges used in different analysis methods, namely power and connectivity. For instance, in power analysis, ‘1H’ refers to the fundamental harmonic, while ‘2H’ represents the second harmonic in response to visual stimuli. In connectivity analysis, θ, α, β, and γ correspond to 4–8 Hz, 8–12 Hz, 12–30 Hz, and 30–50 Hz, respectively. Consequently, a total of 65 EEG-SSVEP features were calculated from measured data utilizing the above equations. This includes 17 lobe power ratio features, 24 lobe connectivity ratio features, and 24 band connectivity ratio features. By utilizing Benjamini–Hochberg (BH) correction and *t*-test filtering methods, eight statistically significant EEG-SSVEP features were selected. These features are described as follows: three lobe power ratio features (5PS-POR-2H, 12PS-POR-1H, and 15PS-POR-1H), two lobe connectivity ratio features (3PS-POR-α and 5PS-POR-γ), and three band connectivity ratio features (3PS-P-ABR, 10PS-P-ABR, and 15PS-P-TBR).

### 2.4. Data Analysis

We employed sequential statistical analysis to assess the differences between the healthy controls and patients with aMCI and validate the performance of behavioral and neurological features. The statistical analysis was conducted using the module ‘statsmodels’ in Python 3.9. First, the chi-squared test and independent samples *t*-test were conducted to compare demographics between healthy controls and patients with aMCI. Second, Kolmogorov–Smirnov tests were conducted to assess normality. Subsequently, when normality was not met, the Wilcoxon signed-rank test was applied. Third, Levene’s test was performed to verify equal variances after confirming normality. If equal variances were confirmed, the independent samples *t*-test was employed. Fourth, in cases where normality was confirmed but unequal variances were observed, Welch’s *t*-test was utilized. Finally, a Pearson correlation analysis was performed to investigate the relationship between behavioral and neurological features. For multiple comparisons of the aforementioned tests and correlation results, we implemented the BH correction [39]. This systematic and structured approach was employed to enhance the reliability of our study’s findings.

### 2.5. Multimodal Learning

In our study, we combined behavioral and neurological features and employed multimodal learning for the early detection of patients suffering from aMCI. All the multimodal learning and embedded methods were carried out using the scikit-learn module in Python 3.9. To identify the best model for behavioral and neurological features, we compared the following six machine learning classifiers: Support Vector Machine (SVM), Linear Discriminant Analysis (LDA), Naive Bayes Classifier (NB), Gaussian Process Classifier (GPC), k-Nearest Neighbors classifier (KNN), and Random Forest (RF) [40,41]. For the feature selection process, an embedded method using SVM with a linear kernel and L2 regularization with C = 0.05 was adopted to filter features with lower importance relative to the average importance across all features [42].

In this study, the dataset consisted of 48 samples in total, with a 7:3 split between training and test data to robustly assess the classifier’s ability to generalize. As a result, 34 samples were allocated for training (17 from healthy controls, 17 from aMCI patients), while the remaining 14 samples were designated for testing (7 from healthy controls, 7 from aMCI patients). To assess overfitting and optimize the model, we used leave-one-out cross-validation (LOOCV), a validation method widely used in medical studies with small sample sizes of fewer than 50 participants [43,44]. This approach allowed us to validate the training data and optimize hyperparameters through grid search (see Table 2). The models underwent a rigorous assessment using various performance metrics, including accuracy, sensitivity, specificity, and the area under the receiver operating characteristic curve (*AUC*). These metrics were calculated as follows with *TP* (true positive), *TN* (true negative), *FP* (false positive), and *FN* (false negative).

(8) $$Accuracy=\frac{TP+TN}{TN+FP+TP+FN}$$

(9) $$Sensitivity=\frac{TP}{TP+FN}$$

(10) $$Specificity=\frac{TN}{TN+FP}$$

(11) $$AUC=\int_{0}^{1}Sensitivity({\left(1-Specificity\right)}^{-1}(x))dx$$

## 3. Results

### 3.1. Differences in Behavioral Features between Healthy Controls and Amnestic Mild Cognitive Impairment Patients

Significant differences in behavioral features between healthy controls and aMCI patients were observed (see Table 3). For aMCI patients, we found several notable differences, including a prolonged time to completion (BH-corrected Wilcoxon signed-rank test, *p* < 0.05), an increased number of errors (BH-corrected Welch’s *t*-test, *p* < 0.05), an extended scanpath length (BH-corrected Wilcoxon signed-rank test, *p* > 0.05), a higher proportion of fixation duration (BH-corrected independent samples *t*-test, *p* < 0.05), a longer hand movement distance (BH-corrected Wilcoxon signed-rank test *p* > 0.05), and a reduced hand movement speed (BH-corrected independent samples *t*-test, *p* < 0.05) compared to healthy controls. These findings resulted from a comprehensive analysis involving various statistical tests, such as the independent samples *t*-test, Welch’s *t*-test, and Wilcoxon signed-rank test, while considering characteristics related to normality and variance. The reported *p*-values were corrected for multiple comparisons using the BH method.

### 3.2. Differences in Neurological Features between Healthy Controls and Amnestic Mild Cognitive Impairment Patients

After analyzing the EEG-SSVEP data using an independent samples *t*-test, aMCI patients showed significant differences in all eight neurological features (see Table 4). Specifically, there were significant differences discerned in 5PS-POR-2H (BH-corrected independent samples *t*-test, *p* < 0.01), 12PS-POR-1H (BH-corrected independent samples *t*-test, *p* < 0.001), and 15PS-POR-1H (BH-corrected independent samples *t*-test, *p* < 0.001) of the lobe power ratio; 3PS-POR-α (BH-corrected independent samples *t*-test, *p* < 0.0001) and 5PS-POR-γ (BH-corrected independent samples *t*-test, *p* < 0.05) in the lobe connectivity ratio; and 3PS-P-ABR (BH-corrected independent samples *t*-test, *p* < 0.05), 10PS-P-ABR (BH-corrected Student’s *t*-test, *p* < 0.05), and 15PS-P-TBR (BH-corrected independent samples *t*-test, *p* < 0.01) of the band connectivity ratio. All reported *p*-values were subjected to correction for multiple comparisons using the BH method.

### 3.3. Correlation between Behavioral and Neurological Features

**Figure 3:**
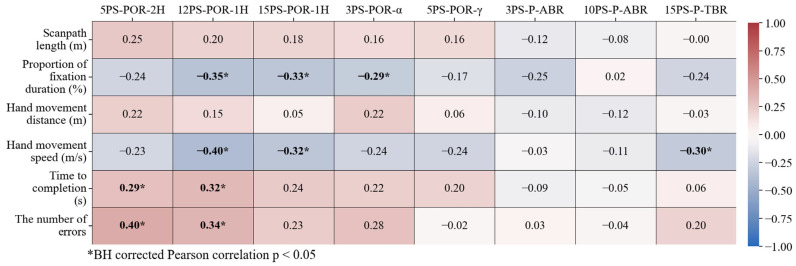
The correlation between VR (vertical axis) and EEG-SSVEP (horizontal axis) features.

In this study, we investigated the relationship between behavioral and neurological features by conducting a Pearson correlation analysis on six selected behavioral features and eight neurological features (see Figure 3). The Pearson correlation analysis revealed significant relations between three behavioral features obtained from the virtual kiosk test (i.e., proportion of fixation duration, hand movement speed, and the number of errors) and two neurological features derived from EEG-SSVEP data (i.e., 5PS-POR-2H, 12PS-POR-1H). Notably, neurological features associated with the dorsal stream showed noteworthy correlations with behavioral features: the lobe power ratio, 5PS-POR-2H, correlated with time to completion (r = 0.29, BH-corrected *p* < 0.05) and the number of errors (r = 0.40, BH-corrected *p* < 0.05). Similarly, 12PS-POR-1H correlated with the proportion of fixation duration (r = −0.35, BH-corrected *p* < 0.05), hand movement speed (r = −0.40, BH-corrected *p* < 0.05), time to completion (r = 0.32, BH-corrected *p* < 0.05), and the number of errors (r = 0.35, BH-corrected *p* < 0.05).

### 3.4. Multivariate Statistical Analysis of Healthy Controls and Patients with Amnestic Mild Cognitive Impairment

**Figure 4:**
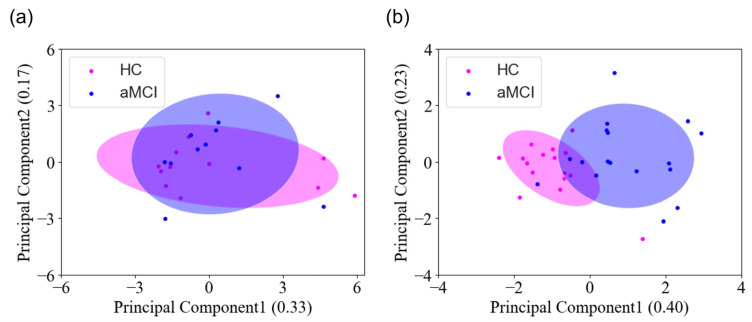
The PCA results between the HC and aMCI groups. (**a**) Results before applying the embedded method, comprising six VR and eight EEG-SSVEP features. (**b**) Results after applying the embedded method, using one VR and four EEG-SSVEP features.

To explore the potential differentiation between healthy controls and individuals with aMCI, we employed PCA as an unsupervised technique (refer to Figure 4). Before the application of the embedded method, Figure 4a demonstrates the overlap between these groups using VR and EEG-SSVEP features, indicating challenges in distinguishing aMCI. Conversely, as depicted in Figure 4b, the embedded method identified five features, encompassing behavioral measures, such as time to completion, and neurological parameters including 15PS-POR-1H, 3PS-POR-α, 3PS-P-ABR, and 15PS-P-TBR, which exhibit the potential to detect aMCI at an early stage.

### 3.5. Multimodal Learning Performance Using Both Behavioral and Neurological Features

In this study, we ultimately selected five features through the embedded feature selection method. Specifically, one was chosen from the behavioral features recorded during the VR test (i.e., time to completion) and four were selected from neurological features (i.e., 15PS-POR-1H, 3PS-POR-α, 3PS-P-ABR, and 15PS-P-TBR).

As illustrated in Table 5, the SVM showed the best performance of our six models, achieving an accuracy of 98.38%, sensitivity of 96.54%, specificity of 100%, and an AUC of 99.73%. To identify the contribution of each of the individual features to the SVM performance, we performed a feature importance analysis. Here, 15PS-P-TBR emerged as the most significant contributor, achieving a feature importance (FI) score of 0.35, followed by 3PS-POR-α (FI = 0.31), 15PS-POR-1H (FI = 0.30), 3PS-P-ABR (FI = 0.29), and time to completion (FI = 0.14).

### 3.6. Comparative Analysis of Early Amnestic Mild Cognitive Impairment Detection

We compared the classification outcomes of our unimodal models using VR and EEG-SSVEP features, respectively, to a multimodal model using both VR and EEG-SSVEP features. The multimodal model using the combined features exhibited the highest performance with 98.38% accuracy, 96.54% sensitivity, 100.00% specificity, and 99.38% AUC. The unimodal model using EEG-SSVEP features only (i.e., 15PS-POR-1H, 3PS-POR-α, 3PS-P-ABR, and 15PS-P-TBR) was ranked second with 93.33% accuracy, 85.71% sensitivity, 100% specificity, and 93.07% AUC. The unimodal model using VR features only (i.e., time to completion) achieved the lowest performance with 53.74% accuracy, 56.28% sensitivity, 51.52% specificity, and 21.92% AUC. These findings are illustrated in Table 6.

## 4. Discussion

The primary objective of this study was to integrate behavioral features derived from VR and neurological features collected from EEG-SSVEP to understand their relationship with cognitive decline and improve the accuracy of detection of aMCI. Looking at behavioral features, aMCI patients exhibited a significantly longer scanpath length, lower proportion of fixation duration, extended hand movement distance, slower movement speed, longer time to completion, and a higher number of errors compared to healthy controls. With respect to neurological features, aMCI patients showed fewer harmonic response and band connectivity differences between the occipital and parietal lobes. A correlation analysis between behavioral and neurological data revealed that the features 5PS-POR-2H and 12PS-POR-1H, which stimulate the α range in the lobe power ratio assessing the response to visual stimuli, showed the strongest correlation with behavioral data including the proportion of fixation duration, hand movement speed, and the number of errors. Showcasing the aMCI early detection performance, an SVM using both VR and EEG-SSVEP features achieved remarkable outcomes: an accuracy of 98.38%, a sensitivity of 96.54%, a specificity of 100%, and an AUC of 99.73%. This SVM model outperformed models using either VR or EEG-SSVEP features alone, with the EEG-SSVEP model achieving an accuracy of 93.33% and the VR feature model attaining an accuracy of 53.74%. Despite the lower performance of the VR feature model, integrating the VR feature (i.e., time to completion) with EEG-SSVEP features improved the model’s performance by 5 percent point. This finding suggests that the inclusion of a VR feature (i.e., time to completion) complements the information on behavioral deficits not captured by EEG-SSVEP features, facilitating a comprehensive assessment of the multifaceted cognitive impairments in aMCI patients. These results underscore the potent capabilities of multimodal analysis, combining behavioral and neurological features, for the early detection of aMCI.

Our findings highlight the significance of understanding the relationship for cognitive decline between the behavioral features, obtained from a VR test environment, and the neurological features, derived from EEG-SSVEP data. Specifically, EEG-SSVEP features (i.e., 5PS-POR-2H, 12PS-POR-1H, and 15PS-POR-1H) computed from power analysis exhibited notable correlations with VR features (i.e., proportion of fixation duration, hand movement speed, time to completion, and the number of errors). These findings align well with previous research that investigated the impact of compromised the dorsal stream on both cognitive function [31,32] and behavioral changes [28,29,30]. For instance, some EEG-SSVEP features obtained from the α range between 8 Hz and 12 Hz, such as 5PS-POR-2H or 12PS-POR-1H, showed decreased power in patients with aMCI. These patients also showed an increased number of errors and slower hand movement speed. Prior studies have demonstrated that impairment of the occipital lobe, the primary hub for visual processing, impedes the effective activation of the pulvinar nucleus, a key facilitator of visual processing acceleration. This impediment leads to challenges in processing visual perception along the ventral stream and, subsequently, action processing along the dorsal stream, thereby exacerbating delays in both perceiving and responding to multiple objects [45,46,47]. Consequently, a compromised visual pathway to the primary visual cortex in the occipital lobe is associated with inadequate acquisition of recognition information for behavior at the dorsal stream, potentially leading to behavioral alterations [21,45,46,47,48,49].

This study is subject to several limitations, which are constraints in collecting medical data, resulting in a restricted sample size. Despite these limitations, it is noteworthy that we have found correlations between behavioral and neurological features and that they perform well when integrated. We have successfully applied multimodal learning to the detection of aMCI and compared the performance of six machine learning models popular in this field. Table 6 shows that a model that combines both behavioral and neurological data outperforms similar models that employ one type of data only. Although the necessity of integrating VR features may seem uncertain given the lower performance (53.74% accuracy) of the model using a single VR feature (i.e., time to completion) compared to the unimodal model using EEG-SSVEP features (93.33% accuracy), it is crucial to note that performance improves to 77.58% accuracy when employing all six VR features. Empirical analysis revealed that the VR feature ‘time to completion’ is the most effective for enhancing the overall performance of the final model integrating both VR and EEG-SSVEP features. Our integrated approach that combined five selected behavioral and neurological features yielded remarkable results, including an accuracy of 98.38%, a sensitivity of 96.54%, a specificity of 100%, and an AUC of 99.73%. Our findings indicate that EEG-SSVEP monitors the processing of visual stimuli in the dorsal stream, whereas VR measures behavior in terms of cognitive functioning. As both methods focus on complementary aspects, their combination into a single model is a powerful tool to monitor and predict behavioral changes. To summarize, our study demonstrated that behavioral changes associated with a compromised dorsal stream can be detected by combining VR and EEG-SSVEP features, leading to improved performance in early aMCI detection through multimodal learning.

## 5. Conclusions

The findings of this study demonstrated that early aMCI detection using both behavioral and neurological features is promising, with 98.38% accuracy, 96.54% sensitivity, 100.00% specificity, and 99.38% AUC. Furthermore, this study established a strong relationship between the behavioral features assessed in the virtual kiosk test and the neurological features measured through EEG-SSVEP. This underscores the importance of integrating multimodal features for the detection of aMCI. Specifically, observations from the virtual kiosk test, including the proportion of fixation duration, hand movement speed, and the number of errors, were found to be closely associated with stimulus responses within the α band range of the lobe power ratio, which account for the dorsal stream stimulus responses. These findings highlight the relationship between VR and EEG-SSVEP features, contributing to understanding how impaired neural responses in the dorsal stream may manifest as behavioral changes. Furthermore, EEG-SSVEP provided insights into the visual stimulus aspects of dorsal stream neural activity, while VR offered behavioral information based on cognitive processing, thus complementing each other in capturing behavioral changes from various perspectives. This synergy resulted in superior performance through SVM-based multimodal learning. Therefore, our multimodal learning approach provides valuable insights into enhancing the performance of early MCI detection by integrating diverse features.

## Figures and Tables

**Table 1:** Demographic and neuropsychological test results of healthy controls and patients with amnestic mild cognitive impairment.

Characteristic	Healthy Controls (*n* = 24)		aMCI ^1^ Patients (*n* = 24)		*p*-Value
	Mean	SD	Mean	SD	
Demographics					
Gender (Male/Female)	10/14		12/12		0.56
Age	68.42	9.92	71.6	7.59	0.22
Years of education	12.13	4.39	10.67	5.71	0.33
Neuropsychological test result					
K-MMSE ^2^	28.30	1.40	27.01	2.38	<0.05

^1^ aMCI: amnestic mild cognitive impairment. ^2^ K-MMSE: Korean Mini-Mental State Examination.

**Table 2:** Overview of hyperparameter optimization outcomes obtained through grid search for improving the performance.

Classifier Models	Hyperparameters
Support Vector Machine	kernel = linear; C = 0.05; probability = true
Linear Discriminant Analysis	solver = singular value decomposition; shrinkage = no shrinkage; tolerance = 1 × 10^−4^
Naive Bayes	priors = none; variance smoothing = 1 × 10^−9^
Gaussian Process	radial basis function (1.0)
K-Nearest Neighbor	k = 5; metric = Euclidean; weights = uniform
Random Forest	number of estimators = 50; max depth = 20; min sample leaf = 4; min sample split = 5

**Table 3:** Comparative statistical analysis of six behavioral features between healthy controls and aMCI patients.

Behavioral Features	Healthy Controls (*n* = 24)		aMCI ^1^ Patients (*n* = 24)		*p*-Value
	Mean	SD	Mean	SD	
Eye movement					
Scanpath length (m)	30.59	37.55	52.60	57.42	>0.05 ^2^
Proportion of fixation duration (%)	56.31	13.98	45.76	16.42	<0.05 ^3^
Hand movement					
Hand movement distance (m)	11.85	6.78	15.95	11.23	>0.05 ^2^
Hand movement speed (m/s)	0.23	0.07	0.18	0.06	<0.05 ^3^
Performance					
Time to completion (s)	50.00	54.94	91.75	84.38	<0.05 ^2^
The number of errors	1.75	1.65	3.50	2.90	<0.05 ^4^

^1^ aMCI: amnestic mild cognitive impairment. ^2^ Wilcoxon signed-rank test *p*-value with Benjamini–Hochberg correction. ^3^ Independent sample *t*-test *p*-value with Benjamini–Hochberg correction. ^4^ Welch’s *t*-test *p*-value with Benjamini–Hochberg correction.

**Table 4:** Statistics from eight neurological features for healthy controls and patients with amnestic mild cognitive impairments.

Neurological Features ^1^	Healthy Controls (*n* = 24)		aMCI ^2^ Patients (*n* = 24)		*p*-Value ^3^
	Mean	SD	Mean	SD	
Lobe Power Ratio					
5PS-POR-2H	0.74	0.08	0.79	0.05	<0.01
12PS-POR-1H	0.72	0.06	0.82	0.09	<0.001
15PS-POR-1H	0.74	0.07	0.81	0.07	<0.001
Lobe Connectivity Ratio					
3PS-POR-α	0.95	0.05	1.02	0.05	<0.0001
5PS-POR-γ	0.99	0.02	1.01	0.04	<0.05
Band Connectivity Ratio					
3PS-P-ABR	0.98	0.08	1.07	0.14	<0.05
10PS-P-ABR	1.04	0.11	1.13	0.17	<0.05
15PS-P-TBR	0.92	0.11	1.04	0.11	<0.01

^1^ Hz frequency of photic stimulation-target lobe-specific frequency range. ^2^ aMCI: amnestic mild cognitive impairment. ^3^ Independent samples *t*-test *p*-value with Benjamini–Hochberg correction.

**Table 5:** Performance of the six classifiers using one behavioral feature and four neurological features.

Classifiers	Accuracy (%)		Sensitivity (%)		Specificity (%)		AUC ^1^ (%)	
	Mean	SD	Mean	SD	Mean	SD	Mean	SD
SVM ^2^	98.38	2.86	96.54	6.12	100.00	0.00	99.73	0.99
KNN ^3^	97.78	3.55	97.40	6.54	98.11	4.48	99.51	1.26
NB ^4^	96.97	3.32	93.51	7.11	100.00	0.00	99.78	1.22
LDA ^5^	93.33	1.64	86.15	2.45	99.62	2.14	93.56	1.52
GPC ^6^	89.70	4.05	87.45	4.66	91.67	6.65	98.27	1.28
RF ^7^	76.16	3.29	87.45	4.66	66.29	5.74	90.69	3.09

^1^ AUC: area under the receiver operating characteristic curve. ^2^ SVM: Support Vector Machine. ^3^ KNN: K-Nearest Neighbors. ^4^ NB: Naive Bayes. ^5^ LDA: Linear Discriminant Analysis. ^6^ GPC: Gaussian Process. ^7^ RF: Random Forest.

**Table 6:** Comparison of aMCI detection based on VR features only, EEG-SSVEP features only, or based on the combination of both features.

Features	Accuracy (%)		Sensitivity (%)		Specificity (%)		AUC ^1^ (%)	
	Mean	SD	Mean	SD	Mean	SD	Mean	SD
VR	53.74	6.95	56.28	42.49	51.52	49.98	21.92	2.50
EEG-SSVEP	93.33	0.00	85.71	0.00	100.00	0.00	93.07	0.58
VR + EEG-SSVEP	98.38	2.86	96.54	6.12	100.00	0.00	99.73	0.99

^1^ AUC: area under the receiver operating characteristic curve.

## Data Availability

The data presented in this study are available on request from the corresponding author. The data are not publicly available due to privacy.
